# Does Gender Influence Colour Choice in the Treatment of Visual Stress?

**DOI:** 10.1371/journal.pone.0163326

**Published:** 2016-09-20

**Authors:** Miriam L. Conway, Bruce J. W. Evans, Josephine C. Evans, Catherine M. Suttle

**Affiliations:** 1 Division of Optometry and Visual Science, School of Health Sciences, City, University of London, London, United Kingdom; 2 Neville Chappell Research Clinic, Institute of Optometry, London, United Kingdom; University of Akron, UNITED STATES

## Abstract

**Purpose:**

Visual Stress (VS) is a condition in which words appear blurred, in motion, or otherwise distorted when reading. Some people diagnosed with VS find that viewing black text on white paper through coloured overlays or precision tinted lenses (PTLs) reduces symptoms attributed to VS. The aim of the present study is to determine whether the choice of colour of overlays or PTLs is influenced by a patient’s gender.

**Methods:**

Records of all patients attending a VS assessment in two optometry practices between 2009 and 2014 were reviewed retrospectively. Patients who reported a significant and consistent reduction in symptoms with either overlay and or PTL were included in the analysis. Overlays and PTLs were categorized as stereotypical male, female or neutral colours based on gender preferences as described in the literature. Chi-square analysis was carried out to determine whether gender (across all ages or within age groups) was associated with overlay or PTL colour choice.

**Results:**

279 patients (133 males and 146 females, mean age 17 years) consistently showed a reduction in symptoms with an overlay and were included. Chi-square analysis revealed no significant association between the colour of overlay chosen and male or female gender (Chi-square 0.788, p = 0.674). 244 patients (120 males and 124 females, mean age 24.5 years) consistently showed a reduction in symptoms with PTLs and were included. Chi-square analysis revealed a significant association between stereotypical male/female/neutral colours of PTLs chosen and male/female gender (Chi-square 6.46, p = 0.040). More males preferred stereotypical male colour PTLs including blue and green while more females preferred stereotypical female colour PTLs including pink and purple.

**Conclusions:**

For some VS patients, the choice of PTL colour is influenced not only by the alleviation of symptoms but also by other non-visual factors such as gender.

## Introduction

Visual stress (VS) is a condition in which text may appear distorted, blurred or in motion when reading, with or without headache [1]. The prevalence of VS is unclear perhaps due to varied criteria used for diagnosis [2] with the proportions of people with VS in unselected samples ranging from less than 5% to over 50% [3].

Some people with VS report that viewing black text on white paper through either coloured overlays or tinted lenses (‘precision tinted lenses’, or PTLs) helps to reduce symptoms, increasing reading speed [4] and accuracy [5]. In 2008 a systematic review and meta-analysis concluded that the use of coloured overlays did not lead to a clear improvement in reading ability and due to lack of evidence could not draw firm conclusions on whether coloured overlays improve symptoms of VS that may be associated with reading disability [6], while a more recent systematic review found that larger, more rigorous randomised controlled trials of interventions for VS are needed [7]. It is important to note that coloured overlays and PTLs aim to reduce the symptoms of VS, rather than to treat the cause of reading difficulty.

Two main theories have been proposed to explain the aetiology of VS and why colour might help alleviate symptoms. The first of these relates to a possible link between dyslexia and a magnocellular pathway deficit, which may lead to unsteady binocular fixation and visual perceptual instability [8, 9]. However, there is evidence indicating that people with VS do not have magnocellular dysfunction [10, 11]. A second theory is based on the idea that cortical cells can in susceptible individuals be over-excited by certain types of stimulation and that coloured overlays or tinted lenses can redistribute the activity within the visual cortex to reduce the amount of excitation [12].

Both of these hypotheses have a physiological basis. However, it is possible that alleviation of symptoms with coloured overlays or tints is due in part to other factors, including placebo effects, attributional reasons (individuals who are struggling with reading may wish to have a visible sign that their difficulties are attributable to a reason other than academic ability), “favourite colour”, photophobia, or longitudinal chromatic aberration [13]. The recommended clinical protocol of excluding optometric anomalies before trying coloured filters [14] and undergoing a trial with coloured overlays before prescribing coloured lenses [15] is designed to minimise these confounding factors. The purpose of the present research is to investigate one of these potential motivations for choosing coloured filters. Specifically, stereotypical gender preferences for colours may influence the choice of colour in some cases.

There is a large body of research into factors influencing colour preference in humans, such as gender and age [16], culture [17] and personality [18]. One of the most widely documented factors is gender. There is a general preference for blue among both males and females in young Western populations [19], but several studies have demonstrated a greater preference among males for blue and females for pink [17,19–21], with female preference for pink and male rejection of pink appearing at around two years of age [22]. These gender-based colour preferences may not exist in non-Western populations since gender-based colour preference varies with culture. For example, previous studies have found that colour preferences differ between Arabic and English populations, at least among females [23], and between industrialised (British) and non-industrialised (Namibian) cultures [24]. Gender-based colour preference may also differ between older and younger populations [25–27], with one study finding that black is the dominant colour choice among teenagers [28]. Thus, gender-based colour preference is complicated by factors such as age and culture.

Regardless of the proposed explanations for the perceived benefits of coloured overlays and PTLs in VS based on visual cortical function, outlined above, there are likely in addition to be a range of potential non-visual factors that may influence colour choice, such as gender. The aim of this study is to determine the extent to which the colour of overlays or PTLs for the alleviation of symptoms in VS is associated, if at all, with gender. For this purpose, we categorised overlay and PTL colour in terms of the gender preference outlined above, with pink and purple colours as ‘female’, blue colours as ‘male’ and other colours ‘neutral’.

## Materials and Methods

Prior to data collection, this research was approved by the City University London School of Health Sciences ethics committee. Records of all patients attending a VS assessment in a community optometry practice in Essex from 2009–2014 and a University Optometry practice in London from 2010–2014 were reviewed retrospectively. All patients had a full orthoptic assessment and eye examination consisting of symptoms and history, vision, cover test, dissociation tests, Mallett aligning prism, ocular motility examination, pupil reactions, near point of convergence, assessment of accommodation (amplitude and MEM), stereopsis, motor fusion, refraction (under cycloplegia where necessary) and fundus examination. After any refractive, binocular vision or accommodative anomalies had been corrected, patients with signs of VS selected Intuitive Overlays in accordance with the procedure described by Wilkins [29]. Any patients showing inconsistent responses to the overlays were re-tested at a later date. Those patients who reported a significant reduction in symptoms were offered an overlay of their preferred colour to take home and use unprompted for several weeks, or longer if necessary. At the end of this period, only those patients who reported a sustained benefit of voluntary overlay use were subsequently tested with the Intuitive Colorimeter in accordance with previously described protocol [30]. The testing methodology in both practices was the same.

For the purposes of the present research, overlays and PTLs were categorized based on gender preferences as described in the literature for Western populations. Specifically, overlay colours in the range of Intuitive Overlays that include colours ‘rose’, ‘pink’ or ‘purple’ were categorised as ‘Female’ on the basis of previous research demonstrating a greater preference for these colours among females than males [22, 23]. On the same basis, overlays including blue or green were categorised as ‘Male’ colours. Blue-purple was assigned as a ‘male’ colour. Overlay colours including neither blue, green, purple nor pink were categorised as ‘Neutral’, and included single overlays of grey, yellow and orange, and overlay combinations including these colours and not including pink, green or blue. Table 1 shows the range of overlay colours presented to patients, and their gender categorisation.

**Table 1:** Gender stereotypical categorisation (as male, female or gender neutral) of colours of overlays presented to each patient. Where two colours are named (e.g. Pink + Pink, or Yellow + Orange) the colour was formed by two overlays superimposed.

Colour of Overlay	Male/female or neutral colours
Yellow	**neutral**
Yellow + Yellow	**neutral**
Yellow + Orange	**neutral**
Orange	**neutral**
Orange + Orange	**neutral**
Rose	**female**
Rose + Orange	**female**
Rose + Rose	**female**
Pink	**female**
Pink + Rose	**female**
Pink + Pink	**female**
Purple	**female**
Purple + Pink	**female**
Purple + Purple	**female**
Blue	**male**
Blue + Purple	**male**
Blue + Blue	**male**
Aqua	**male**
Aqua + Blue	**male**
Aqua + Aqua	**male**
Mint Green	**male**
Mint Green + Aqua	**male**
Mint Green + Mint Green	**male**
Lime Green	**male**
Lime Green + Mint Green	**male**
Lime Green + Lime Green	**male**
Lime Green + Yellow	**male**
Grey	**neutral**
Grey + Grey	**neutral**

The range of overlay and PTL colours included more stereotypical male colours available in the range of overlays than either female or neutral colours. The proportions of males and females choosing these colours were normalised by dividing the number of males or females choosing each particular colour by the number of stereotypical male, female or neutral colours available and turning these into percentages of the male or female cohort.

Overlay data were grouped according to the patient’s gender and the following age groups: younger children (<12 years; n = 99 male, 88 female), older children (12–17 years; n = 22 male, 36 female) and adults (≥18 years; n = 11 male, 23 female). PTL data were categorised using the same age groups: younger children (<12 years; 57 male, 53 female), older children (21 male, 25 female) and adults (≥18 years; 43 male, 45 female). Data were entered into an Excel spreadsheet for subsequent statistical analysis. Chi-square analyses were carried out to determine whether gender (across all ages or within age groups) was correlated with colour overlay preference.

## Results

### Coloured overlay assessment

Two hundred and thirty eight patients from a community optometry practice in Essex completed the overlay assessment and were subsequently prescribed a coloured overlay or combination of two overlays to use at home and/or school (117 males, 121 females, mean age 11.1 years; SD 7.2 years; range 7 to 60). Forty one patients from City University’s optometry clinic completed the overlay assessment and were subsequently prescribed overlays (16 males, 25 females, mean age 23 years; SD 14.5 years; range 7 to 65). Before combining the data sets, they were plotted separately to determine whether they were following the same trends in terms of gender and colour choice. After confirming this they were combined to give a total of 279 patients; 133 males and 146 females, mean age 17 years.

**Fig 1:**
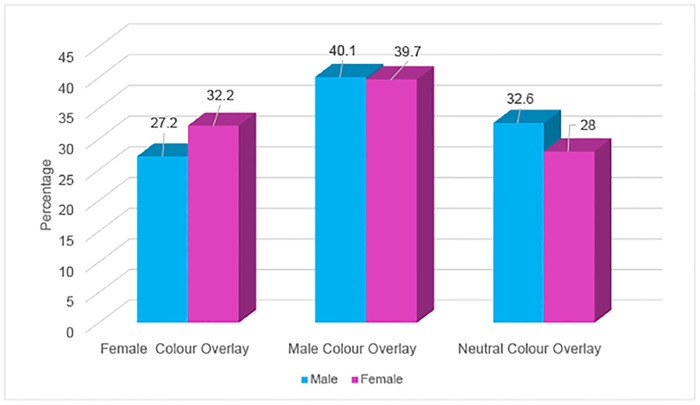
The percentage of male or female patients choosing a stereotypical female, male or neutral overlay in proportion to the number of stereotypical male, female and gender neutral colours available. Percentages were normalised to the total number of available colours in each category.

Chi-square analysis was carried out and revealed that there was no significant association between the colour of overlay chosen between male and female genders (Chi-square 0.788, p = 0.674), as shown by Fig 1 (data included in supplementary file). Chi-square analysis also revealed that there was no significant association between the colour of overlay chosen and male and female genders within each of the three age groups (p>0.15).

### Precision tinted lens assessment

**Fig 2:**
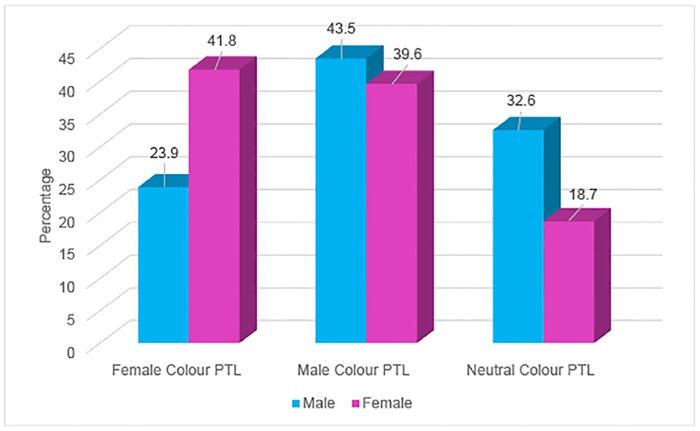
The percentage of male or female patients choosing a stereotypical female, male or neutral PTL in proportion to the number of stereotypical male, female and gender neutral colours available. Percentages were normalised to the total number of available colours in each category.

Two hundred and twenty six patients from a private optometry practice in Essex who found the coloured overlay(s) beneficial completed the colorimetry assessment (PTL) and were subsequently prescribed tinted spectacles. In total there were 113 males and 113 females (mean age 20.5 years; SD 16.2 years; range 7 to 64). Eighteen patients from City University’s Fight for Sight clinic reported sustained benefit with overlays, completed the colorimetry assessment and were subsequently prescribed tinted lenses. In total there were seven males and 11 females (mean age 28.4 years; SD 18.8 years; range 7 to 68). After confirming that the two data sets followed the same trends in terms of gender and colour choice, they were combined to give a total of 244 patients (120 males and 124 females). One third of the sample who were initially prescribed an overlay to use by either practice went on to have PTLs prescribed. Consistent with previous findings [31], over half (54%) of patients who underwent both overlay and PTL assessment chose different colours for each of these (e.g. orange overlay and blue PTL). Chi-square analysis revealed that there was a significant association between the proportion of stereotypical male/female/neutral colours of PTLs chosen and male/female gender (Chi-square 6.46, p = 0.040), as shown by Fig 2. Cramer’s V statistic was 0.163 which indicates a small to medium effect size. More males preferred PTL colours that are not stereotypically female (‘male’ colours such as blue, or neutral colours) while more females preferred stereotypical female colour PTLs including pink and purple. When the group size was broken down into smaller age groups Chi-square analysis revealed that there was no significant association between the colour of PTL chosen, as grouped in this research, and male and female genders within any of the three age groups (p>0.15).

In view of the previously documented general preference for blue among both males and females in young populations,[19] we also analysed our data based on preference for pink or purple versus all other colours. Our results confirmed the above analysis, with a significant association with gender for PTLs (Chi square p = 0.037) but not for coloured overlays (Chi square p = 0.4).

## Discussion

If the choice of overlays and tinted lenses is based purely on the reduction of symptoms attributed to VS then gender should play no role in the choice of either overlay or PTLs. However, it seems more likely that VS is only one of several potential reasons why individuals may choose an overlay. Some of these reasons are listed in the introduction. The usual clinical protocol is that children are issued a coloured overlay and instructed only to return for colorimetry and possible precision tinted lens prescribing if the overlay is found to be beneficial [30] and if the patient is prepared to wear coloured glasses. A marked improvement in reading performance with an overlay is also sometimes taken as justification for testing with the Intuitive Colorimeter [30]. These two criteria (sustained use or performance improvement) are intended to reduce the risk of coloured lenses being prescribed for reasons other than VS. Potentially, one such reason might be “favourite colour”, which seems likely to be gender-related in part. It is therefore surprising that the present research revealed no significant relationship between gender and overlay choice when classified according to stereotypical gender colour preference.

However, the present study did find a tendency for choice of PTL colour to be related to gender in a way that is consistent with previous research studies carried out on adults below the age of 65 years revealing the choice of stereotypical male colours such as blue or green and stereotypical female colours such as pink or purple [17,19,20] (see Fig 2; p = 0.040). One possible explanation for this apparent gender-based preference for lens colour but not for overlay colour is that PTLs are worn by the patient and therefore cosmetic considerations may have a greater influence on the choice of PTLs than overlays. Research has shown that gender moderates the attitude towards colour attractiveness and preference in product choice in both adults [32] and children [33].

In addition, in our sample, the mean age of patients who found overlays beneficial was 17 years, while mean age of those who went on to use PTLs was 24.5 years (p<0.001, unpaired t-test). Perhaps the younger age of the overlay group could account in part for the lack of gender-based colour preference in that group. However, previous research indicating that gender-based colour preferences develop in early childhood and are reduced in adulthood [27] suggests that gender differences in colour preferences would be smaller in our (older) PTL group than in our overlay group, contrary to our findings.

As outlined earlier, preferred colour is associated with other factors including culture. The present study was conducted in a Western, industrialised culture and the colours were categorised accordingly, based on previous research on colour preference in this culture. In different populations, colour categorisation and gender-based preference may differ.

While the colour choices shown in Fig 2 are related to gender, over half (56.5%) of the males represented in this figure chose a colour that is not a stereotypical “male colour” (including ‘female’ or ‘neutral’ colours) and over half (58.3%) of females choose a colour that is not a stereotypical “female colour”. Thus, factors other than gender influence colour choice in this population, and gender makes a relatively minor contribution.

Previous research suggests that PTLs influence the visual cortical response in children [34,35] and adults [36] with VS, and adults with migraine [37,38]. These findings support the notion that PTLs may be effective in moderating cortical activity, but these studies all involved small sample sizes and more research is required to understand any connection between PTL or overlay colour and visual cortical activity in VS. The present findings do not shed light on visual cortical function in VS, but indicate that choice of PTL colour is associated with gender, and that non-visual factors play a part in PTL colour choice.

The use of coloured overlays and PTLs to treat VS in patients with and without reading disability remains controversial, as debate surrounds whether they are effective in the reduction of symptoms due to an impact on an underlying cause such as cortical over-excitation, or merely due to a placebo effect. The effect of colour in VS has been tested in several studies with Intuitive Overlays [4,14,31, 39–45]. Although these studies have all produced positive results, research with coloured overlays cannot fully control for the placebo effect. One study [46] used the Intuitive Colorimeter to compare VS patients’ chosen PTLs with lenses of a very similar (indistinguishable) colour and found that the number of symptom-free days was higher with the PTL than with the comparison tint. The study used symptom diaries and recorded days on which symptoms were present rather than other factors such as the severity of symptoms or the number of symptomatic periods during the day. Another study [31] using the Intuitive Colorimeter found an improvement with the chosen colour but not with a similar control colour. The number of participants who completed both these studies was modest and further randomised controlled trials are needed with larger sample sizes.

In the present study, the statistical significance of the gender difference in PTL choice was small (p = 0.04) despite a fairly large sample size and no significant differences were found within the age groups. This lack of difference in the (smaller) age range groups may be due to the small sample sizes in this sub-group analysis. Thus, to explore whether gender-based choice is stronger in particular age ranges requires a larger sample.

In conclusion our study has shown that, in this sample of VS patients, stereotypical gender-based colour preferences are not associated with the choice of coloured overlay, but are associated with choice of PTL. This finding indicates that for some patients there are factors other than symptoms of VS that are influencing colour choice. The reasons for colour choice are likely to be multifactorial, including personal colour preference; larger studies are needed to understand the extent to which non-visual factors affect overlay and PTL choice.

## Supporting Information

S1 Table Shows gender and age categories alongside overlay colour category for each participant. (PDF)

S2 Table shows gender and age categories alongside PTL colour choice category for each participant. (PDF)

## References

[1] Wilkins A, Nimmo-Smith I, Tait A, McManus C. Della Sala S, Tilley A et al A neurological basis for visual discomfort. *Brain*. 1984;107 (Pt 4):989–1017. 6509314 10.1093/brain/107.4.989

[2] Griffiths PG Using coloured filters to reduce the symptoms of visual stress in children with reading delay. *Scan J Occup Ther* 2015 22(5): 328–329. 10.3109/11038128.2015.1033456 25892164

[3] Kriss I, Evans BJ. The relationship between dyslexia and Meares-Irlen syndrome. *J Res Read*. 2005;28(3):350–364.

[4] Jeanes R, Busby A, Martin J, Lewis E, Stevenson N, Pointon D et al Prolonged use of coloured overlays for classroom reading. *Br J Psychol*. 1997;88(4):531–548. 9415962

[5] Robinson G, Foreman P. Scotopic sensitivity/irlen syndrome and the use of coloured filters: A long-term placebo controlled and masked study of reading achievement and perception of ability. *Percept Mot Skills*. 1999;89(1):83–113. 10544403 10.2466/pms.1999.89.1.83

[6] Albon E, Adi Y, Hyde C, West Midlands Health Technology Assessment Collaboration. *The effectiveness and cost-effectiveness of coloured filters for reading disability*: *A systematic review*. West Midlands Health Technology Assessment Collaboration, Department of Public Health and Epidemiology, University of Birmingham; 2008.

[7] Evans BJ, Allen PM (2016) A systematic review of controlled trials on visual stress using intuitive overlays or the intuitive colorimeter. *J Optom* In press. 10.1016/j.optom.2016.04.002 PMC5030324 27425262

[8] Stein J. The magnocellular theory of developmental dyslexia. *Dyslexia*. 2001;7(1):12–36. 11305228 10.1002/dys.186

[9] Nandakumar K, Leat SJ (2008) Dyslexia: a review of two theories. *Clin Exp Optom* 91(4): 333–340. 10.1111/j.1444-0938.2008.00277.x 18430036

[10] Simmers AJ, Bex PJ, Smith FK, Wilkins AJ (2001) Spatiotemporal visual function in tinted lens wearers. *Invest Ophthalmol Vis Sci* 42(3): 879–884. 11222554

[11] White S, Milne E, Rosen S, Hansen P, Swettenham J, Frith U, Ramus F. (2006) The role of sensorimotor impairments in dyslexia: a multiple case study of dyslexic children. *Dev Sci* 9(3): 237–255. 16669791 10.1111/j.1467-7687.2006.00483.x

[12] Huang J, Zong X, Wilkins A, Jenkins B, Bozoki A, Cao Y fMRI evidence that precision ophthalmic tints reduce cortical hyperactivation in migraine. *Cephalalgia*. 2011 31(8):925–36. 10.1177/0333102411409076 21622479 PMC3132147

[13] Scott L, McWhinnie H, Taylor L, Stevenson N, Irons P, Lewis E et al Coloured overlays in schools: Orthoptic and optometric findings. *Ophthalmic Physiol Opt*. 2002;22(2):156–165. 12014489 10.1046/j.1475-1313.2002.00009.x

[14] Lightstone A, Evans B. A new protocol for the optometric management of patients with reading difficulties. *Ophthalmic Physiol Opt*. 1995;15(5):507–512. 8524584

[15] Allen PM, Hussain A, Usherwood C, Wilkins A. Pattern-related visual stress, chromaticity, and accommodation. *Invest Ophthalmol Vis Sci*. 2010;51(12):6843–6849. 10.1167/iovs.09-5086 20610842

[16] Chiu SW, Gervan S, Fairbrother C, Johnson L, Owen-Anderson A, Bradley S. Sex-dimorphic color preference in children with gender identity disorder: A comparison to clinical and community controls. *Sex Roles*. 2006;55(5–6):385–395.

[17] Sorokowski P, Sorokowska A, Witzel C. Sex differences in color preferences transcend extreme differences in culture and ecology. *Psychon Bull Rev*. 2014;21(5):1195–1201. 10.3758/s13423-014-0591-8 24570324 PMC4181517

[18] Bjerstedt A. Warm-cool color preferences as potential personality indicators: Preliminary note. *Percept Mot Skills*. 1960;10(1):31–34.

[19] Hurlbert AC, Ling Y. Biological components of sex differences in color preference. *Curr Biol*. 2007;17(16):R623–R625. 17714645 10.1016/j.cub.2007.06.022

[20] Ellis L, Ficek C. Color preferences according to gender and sexual orientation. *Pers Indiv Diff*. 2001;31(8):1375–1379.

[21] Silver NC, McCulley WL, Chambliss LN, Charles CM, Smith AA, Waddell WM et al Sex and racial differences in color and number preferences. *Percept Mot Skills*. 1988;66(1):295–299.

[22] Lobue V, Deloache JS (2011) Pretty in pink: The early development of gender-stereotyped colour preferences. Br Dev Psychol 29(3): 656–667. 10.1111/j.2044-835X.2011.02027.x 21848751

[23] Al-Rasheed AS (2015) An experimental study of gender and cultural differences in hue preference. *Front Psychol* 6:30 10.3389/fpsyg.2015.00030 25688219 PMC4311615

[24] Taylor C, Clifford A, Franklin A Color preferences are not universal. 2013 *J Exp Psychol Gen*. 142(4):1015–1027. 10.1037/a0030273 23148465

[25] Mather J, Stare C, Breinin S. Color preferences in a geriatric population. *Gerontologist*. 1971;11(4 Part 1):311–313. 5127899 10.1093/geront/11.4_part_1.311

[26] Wijk H, Berg S, Sivik L, Steen B. Color discrimination, color naming and color preferences in 80-year olds. *Aging*. 1999;11(3):176–185. 10476313

[27] Wong WI, Hines M (2015) Preferences for pink and blue: The development of colour preferences as a distinct gender-typed behavior in toddlers. *Arch Sex Behav* 44(5): 1243–1254. 10.1007/s10508-015-0489-1 25680819

[28] Akcay O. Marketing to teenagers: The influence of color, ethnicity and gender. *IJBSS*. 2012;3(22).

[29] Wilkins A. A system for precision ophthalmic tinting. *Manual for the Intuitive Colorimeter Mk* 2. 1993.

[30] Allen PM, Evans BJ, Wilkins AJ. Vision and reading difficulties. Ten Alps Creative; 2010.

[31] Lightstone A, Lightstone T, Wilkins A. Both coloured overlays and coloured lenses can improve reading fluency, but their optimal chromaticities differ. *Ophthalmic Physiol Opt*. 1999;19(4):279–285. 10645383 10.1046/j.1475-1313.1999.00442.x

[32] Funk D, Oly Ndubisi N. Colour and product choice: A study of gender roles. *Man res rev*. 2006;29(1/2):41–52.

[33] Auty S, Lewis C. Exploring children's choice: The reminder effect of product placement. *Psychol Market*. 2004;21(9):697–713.

[34] Riddell PM, Wilkins A, Hainline L. The effect of colored lenses on the visual evoked response in children with visual stress. *Optom Vis Sci*. 2006;83(5):299–305. 16699442 10.1097/01.opx.0000216125.83236.af

[35] Chouinard BD, Zhou CI, Hrybouski S, Kim ES, Cummine J. A functional neuroimaging case study of Meares—Irlen syndrome/visual stress (MISViS). *Brain Topogr*. 2012;25(3):293–307. 10.1007/s10548-011-0212-z 22124535

[36] Kim JH, Seo H, Ha S, Kim S. Functional magnetic resonance imaging findings in meares-irlen syndrome: A pilot sudy. *Korean J Ophthalmol*. 2015;29(2):121–125. 10.3341/kjo.2015.29.2.121 25829829 PMC4369514

[37] Huang J, Zong X, Wilkins A, Jenkins B, Bozoki A, Cao Y. fMRI evidence that precision ophthalmic tints reduce cortical hyperactivation in migraine. *Cephalalgia*. 2011;31(8):925–936. 10.1177/0333102411409076 21622479 PMC3132147

[38] Coutts LV, Cooper CE, Elwell CE, Wilkins AJ. Time course of the haemodynamic response to visual stimulation in migraine, measured using near-infrared spectroscopy. *Cephalalgia*. 2012;32(8):621–629. 10.1177/0333102412444474 22623757

[39] Wilkins A, Jeanes R, Pumfrey P, Laskier M. Rate of reading test^®^: Its reliability, and its validity in the assessment of the effects of coloured overlays. *Ophthalmic Physiol Opt*. 1996;16(6):491–497. 8944196

[40] Wilkins A, Lewis E, Smith F, Rowland E, Tweedie W. Coloured overlays and their benefit for reading. *J Res Read*. 2001;24(1):41–64.

[41] Bouldoukian J, Wilkins AJ, Evans BJ. Randomised controlled trial of the effect of coloured overlays on the rate of reading of people with specific learning difficulties. *Ophthalmic Physiol Opt*. 2002;22(1):55–60. 11829008 10.1046/j.1475-1313.2002.00002.x

[42] Northway N. Predicting the continued use of overlays in school children—a comparison of the developmental eye movement test and the rate of reading test. *Ophthalmic Physiol Opt*. 2003;23(5):457–464. 12950892 10.1046/j.1475-1313.2003.00144.x

[43] Hollis J, Allen PM. Screening for Meares—Irlen sensitivity in adults: Can assessment methods predict changes in reading speed? *Ophthalmic Physiol Opt*. 2006;26(6):566–571. 17040420 10.1111/j.1475-1313.2006.00401.x

[44] Singleton C, Henderson L. Computerized screening for visual stress in children with dyslexia. *Dyslexia*. 2007;13(2):130–151. 17557688 10.1002/dys.329

[45] Allen PM, Gilchrist JM, Hollis J. Use of visual search in the assessment of pattern-related visual stress (PRVS) and its alleviation by colored filters. *Invest Ophthalmol Vis Sci*. 2008;49(9):4210–4218. 10.1167/iovs.07-1587 18469191

[46] Wilkins A, Evans B, Brown J, Busby AE, Wingfield AE, Jeanes RJ, Bald RJ. Double-masked placebo-controlled trial of precision spectral filters in children who use coloured overlays. *Ophthalmic Physiol Opt*. 1994;14(4):365–370. 7845693

