# Supplementary material for: Does Gender Influence Colour Choice in the Treatment of Visual Stress?
Source: PLoS One. 2016 Sep 20;11(9):e0163326. doi: 10.1371/journal.pone.0163326 (PMC5029909; doi:10.1371/journal.pone.0163326)
Supplement: S2 Table — (PDF) [file pone.0163326.s002.pdf]

Gender and age categories shown alongside PTL colour choice category for each participant

Colour	Gender	Age
2	0	1
1	0	3
2	1	1
2	0	1
2	0	2
1	1	1
1	0	3
2	0	3
3	1	1
1	0	3
2	0	1
2	0	1
1	0	1
1	1	1
3	1	1
2	1	1
2	1	1
2	0	3
1	1	1
1	1	3
1	1	1
2	1	3
1	1	1
2	0	3
1	0	2
2	0	3
1	1	2
2	0	2
2	1	2
1	1	2
2	0	2
2	0	2
1	0	1
1	0	1
2	1	3
2	1	1
2	1	2
2	0	1
1	1	3
1	1	3
2	1	1
3	0	1
2	1	3
2	0	3
2	1	2

Colour code
1 = female colour
2 = male colour
3 = neutral colour

Gender code
0 = male
1 = female

Age code
1 = <12 years
2 = 12 to 17 years
3 = ≥ 18 years

2	1	1
2	0	1
1	1	2
2	1	2
2	1	1
1	1	3
2	1	1
2	0	1
3	0	1
1	1	1
1	1	1
3	1	2
2	1	1
2	1	1
1	1	1
2	0	1
2	1	1
2	0	1
2	0	1
1	1	3
2	0	2
2	1	2
1	1	3
2	1	3
3	0	3
2	1	1
2	1	3
2	0	2
2	0	3
2	1	3
2	1	3
2	1	1
1	1	1
2	0	1
1	0	2
2	1	3
2	1	3
2	1	2
2	1	2
2	0	3
2	0	3
2	1	3
2	1	1
1	1	1
2	1	1
2	1	1
1	1	1
3	1	1
1	1	1
2	0	1

2	0	1
1	1	3
2	1	1
2	0	2
3	0	2
2	1	3
1	0	3
1	1	1
2	1	2
1	1	3
2	0	3
1	1	1
2	1	3
2	0	1
2	1	3
2	0	1
2	0	1
1	1	3
2	0	3
2	1	2
1	0	3
1	0	1
2	1	3
1	0	3
2	0	3
2	1	2
2	0	3
2	0	3
2	1	2
2	1	1
2	1	1
2	0	1
2	0	3
2	0	1
1	0	1
2	0	1
1	0	3
2	0	2
2	1	3
2	0	1
2	1	1
2	1	1
2	0	3
2	0	1
2	0	3
1	0	1
2	0	1
2	1	2
2	0	2
1	1	3

1	0	1
2	0	3
1	0	3
2	1	1
2	1	1
1	1	2
2	1	2
2	0	1
2	0	3
1	1	3
1	1	2
2	1	1
1	1	3
2	1	1
1	0	1
2	0	1
2	0	2
2	1	3
1	1	3
1	1	2
2	0	3
2	0	1
2	0	3
2	0	1
2	1	1
2	0	1
2	0	2
2	1	1
1	0	3
2	0	2
1	1	3
2	0	1
2	1	3
2	1	3
2	0	1
2	1	1
1	1	3
2	1	1
2	1	1
2	0	3
2	0	1
2	1	3
2	0	1
1	1	1
2	0	1
2	1	3
2	0	1
2	1	2
2	0	1
1	0	1

2	0	3
2	0	3
2	0	1
2	1	3
2	0	1
2	0	1
1	1	1
2	0	1
2	1	2
2	0	2
1	0	1
2	0	3
2	1	2
2	1	3
2	0	3
1	1	3
3	0	2
3	0	1
2	0	2
3	0	2
2	0	3
2	0	2
2	0	3
2	0	1
2	1	3
1	0	2
1	0	1
2	0	1
2	1	1
2	1	3
3	0	1
1	1	1
1	1	1
2	0	1
1	1	1
1	1	3
1	0	3
2	1	2
2	1	3
3	0	3
2	1	2
2	0	3
3	1	3
2	1	3
1	0	3
2	0	3
1	1	3
2	0	1
2	1	3
